# Frequency of Cushing’s syndrome due to ACTH-secreting adrenal medullary lesions: a retrospective study over 10 years from a single center

**DOI:** 10.1007/s12020-016-1127-y

**Published:** 2016-10-03

**Authors:** Henrik Falhammar, Jan Calissendorff, Charlotte Höybye

**Affiliations:** 1 Department of Endocrinology, Metabolism and Diabetes, Karolinska University Hospital, Stockholm, Sweden; 2 Department of Molecular Medicine and Surgery, Karolinska Institutet, Stockholm, Sweden; 3 Department of Clinical Science and Education, Karolinska Institutet, Södersjukhuset, Stockholm, Sweden

**Keywords:** Cushing, Ectopic, Adrenal medullary hyperplasia, Pheochromocytoma

## Abstract

Cushing’s syndrome due to ectopic adrenocorticotropic hormone production from adrenal medullary lesions has occasionally been described. We retrospectively reviewed all 164 cases of Cushing’s syndrome and 77 cases of pheochromocytomas during 10 years. Of all cases with Cushing’s syndrome, only two cases (1.2 %) were due to ectopic adrenocorticotropic hormone production from adrenal medullary lesions (one case of pheochromocytoma and one case of adrenal medullary hyperplasia). Of all pheochromocytomas only the above-mentioned case (1.3 %) also gave rise to an ectopic adrenocorticotropic hormone syndrome. The clinical presentation of adrenocorticotropic hormone-secreting pheochromocytoma and adrenal medullary hyperplasia can be anything from mild to dramatic. These are rare conditions important to bear in mind in the workup of a patient with Cushing’s syndrome or with pheochromocytoma. The identification of ectopic adrenocorticotropic hormone secretion from adrenal medullary lesions can be life-saving.

## Introduction

Cushing’s syndrome caused by endogenous hypercortisolism is a rare disorder with an incidence of about 0.2–5.0 cases per million population per year and a prevalence of about 39–79 per million [1]. Endogenous Cushing’s syndrome is divided into adrenocorticotropic hormone (ACTH)-dependent, mainly pituitary tumors and ectopic ACTH production, or ACTH-independent, mainly adrenocortical tumor/hyperplasia and adrenocortical cancer [1]. Pheochromocytomas are also rare but in certain groups such as in unselected patients with adrenal incidentaloma 0.6–1.4 % are affected [2, 3]. Both Cushing’s syndrome and pheochromocytoma are serious conditions, which can be fatal if not diagnosed and/or managed appropriately [1, 4]. The combination of both hypercortisolism and increased catecholamine secretion has an even higher fatality potential and has infrequently been reported in the literature in ectopic ACTH syndrome due to pheochromocytoma [5–8].

The aims of the present study were to determine in a large cohort with Cushing’s syndrome from a single center the frequency of hypercortisolism due to ACTH secretion from adrenal medullary lesions and how frequently pheochromocytomas result in ectopic ACTH syndrome. Moreover, we also wanted to characterize the identified cases.

## Subjects and methods

This retrospective study was conducted at the Department of Endocrinology, Metabolism and Diabetes, Karolinska University Hospital, Stockholm, Sweden. All patients with an International Classification of Diseases version 10 (ICD-10) code of E275 (adrenomedullary hyperfunction), C741 (malignant neoplasm of medulla of adrenal gland), E240 (pituitary-dependent Cushing’s disease), E243 (ectopic ACTH syndrome), E248 (other Cushing’s syndrome), and/or E249 (Cushing’s syndrome, unspecified) and who had attended the out-patient clinic between June 2005 and June 2015 were eligible for inclusion. All the electronic medical files of the patients were reviewed manually and those where the final diagnosis did not correspond to the previous mentioned ICD-10 code(s) were subsequently excluded. All specialist out-patient visits in Sweden are coded with ICD-10 codes by the attending physician and are thereafter stored in both local and national databases [9]. The clinical, biochemical, and histological data of patients with Cushing’s syndrome due to ectopic ACTH secretion from the adrenal medulla were recorded in detail.

Cushing’s syndrome was diagnosed if at least two biochemical tests, 1 mg overnight dexamethasone suppression test, 24 h urine cortisol and midnight serum cortisol, were clearly abnormal, together with a clinical picture of Cushing’s syndrome [3]. ACTH-independent (adrenal) Cushing’s syndrome was diagnosed if the ACTH was decreased, otherwise an ACTH-dependent Cushing’s syndrome was presumed. To discriminate between pituitary or ectopic ACTH production, magnetic resonance imaging (MRI) of the pituitary gland, high-dose dexamethasone suppression test, and/or bilateral inferior petrosal sinus sampling were performed [1].

Plasma ACTH was measured on an advantage automatic immune analyzer (Nichols Institute Diagnostics, San Clemente, CA, USA), serum cortisol using electrochemical luminescence Modular E (Roche Diagnostics, Mannheim, Germany), urinary cortisol and plasma metanephrine and normetanephrine using liquid chromatography-tandem mass spectrometry (Sigma-Aldrich, St. Louis, MO, USA), and plasma chromogranin A using radioimmunoassay (Malmö, Sweden).

The Regional Ethical Review Board in Stockholm, Sweden, approved the study.

## Results

In total 164 cases of Cushing’s syndrome were found of which 61.6 % owed to excess ACTH production from a pituitary tumor (Cushing’s disease), 32.3 % to an adrenocortical tumor/hyperplasia, and 6.1 % to ectopic ACTH syndrome (Table 1). Of the 10 patients with ectopic ACTH syndrome, 1 (10 %) was due to adrenal medullary hyperplasia and 1 (10 %) due to a pheochromocytoma. Thus, 20 % of all ectopic Cushing’s syndrome, but only 2 of 164 cases (1.2 %) of all Cushing’s syndromes, were the result of ACTH-secreting adrenal medullary lesions.

**Table 1:** Causes of endogenous Cushing’s syndrome in 164 cases attended out-patient clinic during 10 years at the Karolinska University Hospital

ACTH-dependent, n	111 (67.7 %)	
Cushing’s disease, *n*	101 (61.6 %)	
Ectopic ACTH syndrome, *n*	10 (6.1 %)	
Small cell lung cancer, *n*		3 (30 %)
Bronchial carcinoid, *n*		2 (20 %)
Endocrine pancreas tumor, *n*		1 (10 %)
Cervix cancer, *n*		1 (10 %)
Pregnancy-associated, *n*		1 (10 %)
Pheochromocytoma, *n*		1 (10 %)
Adrenal medullar hyperplasia, *n*		1 (10 %)
Unknown, *n*		1 (10 %)
ACTH-independent	53 (32.3 %)	
Adrenocortal adenoma/hyperplasia	47 (28.7%)	
Adrenocortical cancer	6 (3.6 %)	
Total	164 (100 %)	10 (100 %)

*Note* Special focus has been given to ectopic ACTH syndrome

In total 77 cases of pheochromocytomas had been diagnosed of which the previously mentioned patient (1.3 %) had also given rise to an ectopic ACTH syndrome.

### Case 1

**Fig. 1:**
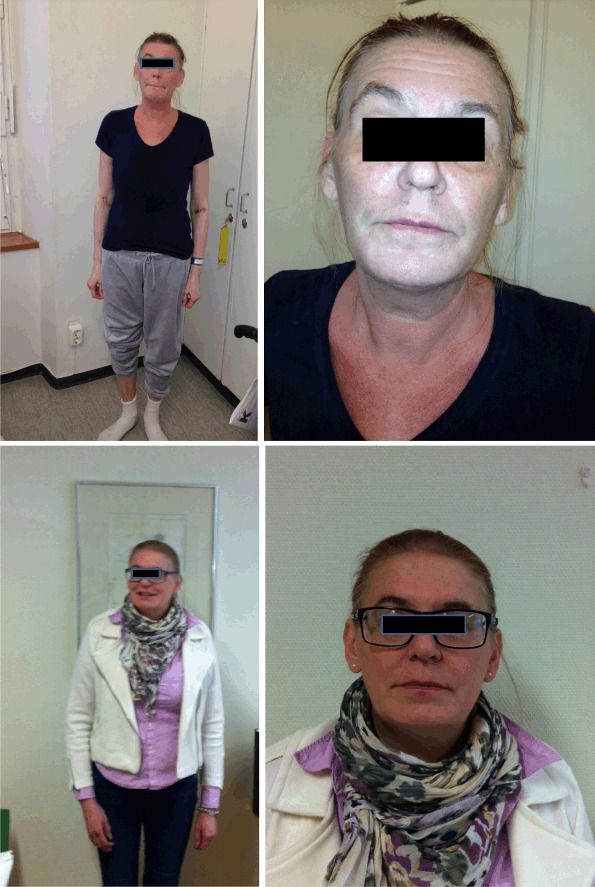
50-year-old women (Case 1) with ectopic ACTH syndrome due to a pheochromocytoma with a rapid weight loss, muscle wasting, hypokalemia, high glucose levels, and hirsutism (*upper panel*). Two months after surgery she had returned to her previous weight and her symptoms had disappeared (*lower panel*)

**Fig. 2:**
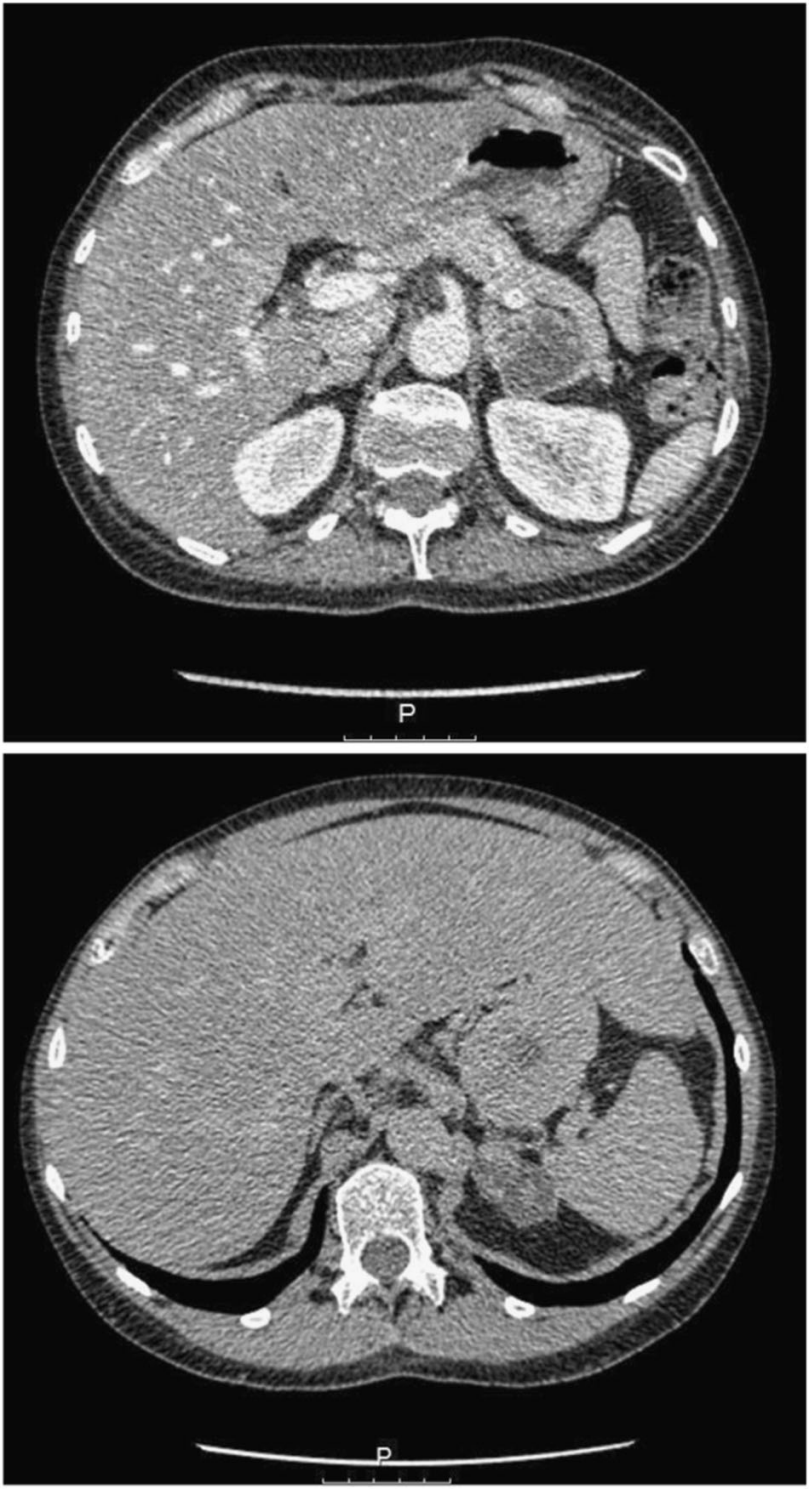
CT with contrast from Case 1 demonstrating a left-sided 3.5 × 4.5 cm large adrenal tumor with peripheral contrast enhancement and septa-like structures in addition to bilateral adrenal hyperplasia (*upper panel*). CT without contrast from Case 2 showing a left-sided 3.5 × 4.5 × 5 cm large heterogeneous adrenal mass (*lower panel*)

**Fig. 3:**
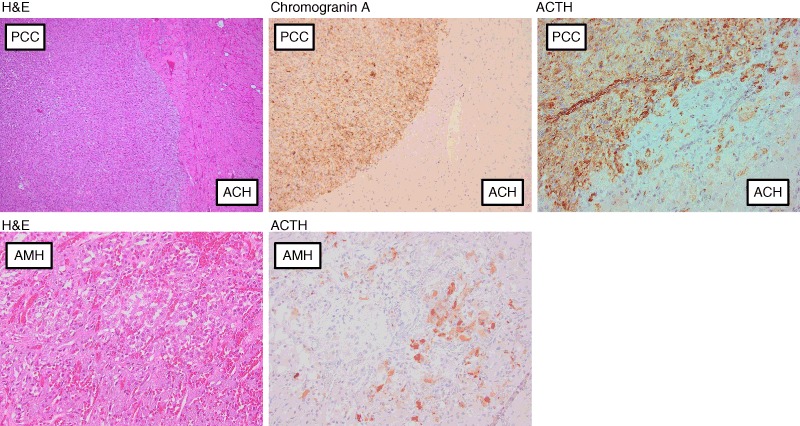
Photomicrographs of the adrenal medullary lesions from Case 1 (*upper panel*) and Case 2 (*lower panel*), respectively. In Case 1, the pheochromocytoma is clearly positive for chromogranin A and ACTH, whereas the surrounding adrenocortical hyperplasia (secondary to the ACTH production) is negative. In Case 2, the photomicrographs represent the adrenal medullary hyperplasia with focal ACTH production. *H&E* hematoxylin-eosin staining, *PCC* pheochromocytoma, *ACH* adrenocortical hyperplasia, *AMH* adrenal medullary hyperplasia

A 50-year-old woman presented to the emergency department (ED) due to gastroenteritis followed by palpitations, headache, and anxiety. She had a previous history of headaches, tremor, chest pain, palpitations, anxiety, and perspiration for at least 3 years and had had both a Coronary Care Unit admission and a neurology out-patient review for these symptoms. There was no family history of Cushing’s syndrome or pheochromocytoma. An increased blood pressure of 160/105 was found with slightly low plasma potassium (3.2 mmol/L). Bisoprolol 2.5 mg was initiated, but as the patient did not feel well on this medication a change to amlodipine 5 mg daily was made with a plan of follow-up with the general practitioner. She presented again to ED a month later with decreased appetite, weight loss of 12 kg during the last month (from 65 to 53 kg), fatigue, muscle weakness, anxiety, hirsutism, increased urine output, blurry vision, and increased palpitation and tremor. Increased plasma glucose levels and severely low potassium were found together with a metabolic alkalosis (Table 2). She was admitted with potassium replacement and insulin treatment. Following days serum cortisol was measured at different times and they were all >2000 nmol/L and urinary cortisol level was very high. Urinary steroid profile demonstrated increased secretion of cortisol metabolites and also androgens consistent with Cushing’s syndrome but no precursor steroids that could indicate an adrenocortical cancer. Her appearance was not typical for Cushing’s syndrome but she looked unhealthy with muscle wasting and rapid weight loss (Fig. 1, upper panel). A computed tomography (CT) scan identified a 3.5-cm large mass on the left adrenal and radiological bilateral adrenal hyperplasia (Fig. 2, upper panel), while the abdomen, thorax, and MRI of the pituitary were normal. Subsequent plasma ACTH was high, plasma metanephrine was almost three times upper reference limit, while plasma normetanephrine was just above the upper reference limit (Table 2). Alfa blocker and spironolactone were added and she underwent an uncomplicated left adrenalectomy 3 weeks after the admission under the cover of hydrocortisone infusion and later oral hydrocortisone. After surgery, the doses of insulin and potassium were decreased dramatically and she was discharged 4 weeks after the admission much improved. Histology showed a radically extirpated 3.5 × 2.2 × 1.5 cm large pheochromocytoma displaying multifocal evidence vascular invasion. The majority (75–100 %) of tumor cells expressed ACTH and the Ki67-index was 7 %. The surrounding adrenocortical tissue was hyperplastic and negative for ACTH immunoreactivity (Fig. 3, upper panel). Since the current WHO criteria for malignancy (presence of metastases) could not be fulfilled, the tumor was considered benign with potential of malignant behavior and so close follow-up was recommended. Two months post surgery she was in a good shape, had regained her previous weight, and all her symptoms had disappeared (Fig. 1, lower panel). During the next 4 months all medications were weaned off and she had started doing exercise on a regular basis. She claimed feeling better than she had done in many years. Three years later, there was still no biochemical or radiologic signs of recurrence.

**Table 2:** Biochemical results pre-adrenalectomy and post-adrenalectomy in two cases with ectopic ACTH syndrome due to pheochromocytoma (Case 1) or adrenal medullary hyperplasia (Case 2)

		Case 1		Case 2		
	Time	Pre-operative	Post-operative	Pre-operative	Post-operative	Normal range
P-potassium (nmol/L)		2.4	3.9	4.0	4.8	3.5–4.4
P-glucose (mmol/L)		33 (random)	4.3	5.0	5.4	4.0–6.0 (fasting)
Base excess (mmol/L)		14.8				−3–3
pH		7.57				7.35–7.45
S-cortisol (nmol/L)	8 am	>2000	<15*	671	<15	200–800
	Midnight	>2000		462		<50
U-cortisol (nmol/24 h)		22400		346		40–170
Low-dose DST (nmol/L)				736		<50
High-dose DST(nmol/L and nmol/24 h)		>2000		993		<50
				5030		<14
P-ACTH (pmol/L)	8 am	149	4	12	<0.5	(2.0–10)
fP-metanephrine (nmol/L)		1.1	0.2	<0.2	<0.2	<0.3
fP-normetanephrine (nmol/L)		0.7	<0.2	<0.2	0.4	<0.6
fP-chromogranin A (nmol/L)		10.4	0.8	1.8		<3.0
P-renin (mU/L)		40				2.8–40
P-aldosterone (pmol/L)		231				<650

*P* plasma, *S* serum, *U* urine, *DST* dexamethasone suppression test, *f* fasting. *505 nmol/L 6 months later without any medication

### Case 2

A 44-year-old woman with levothyroxin-treated hypothyroidism was referred to the out-patient clinic from her general practitioner with the question of Cushing’s syndrome. The last few years she had suffered from depression, fatigue, insomnia, concentration difficulties, easy bruising, proximal muscle weakness, and muscle and joint pain, as well as amenorrhea. Her face had got slightly round and red. Fat deposition at the supraclavicular fossa, between the shoulders and abdomen was noted. Acne and perspiration were present but no hirsutism. Physical fitness had decreased but she was still jogging or cycling a few times a week to keep her weight stable. She had acquired stress fractures to her foot in connection with running. The symptoms had aggravated significantly during the last 3 months before referral. The patient had been on sick leave intermittently during the last years. There was no family history of Cushing’s syndrome or pheochromocytoma. Blood pressure was 130/85. Serum and urine cortisol were increased, while plasma ACTH was only marginally elevated (Table 2). MRI of the pituitary was normal and sinus petrosus sampling could not show an increase in ACTH after corticotropin-releasing hormone (CRH) stimulation. Low-dose and high-dose dexamethasone suppression test did not suppress cortisol levels, but instead paradoxical increases were seen. CT of the neck, thorax, and abdomen revealed a heterogeneous 5-cm large left adrenal mass (Fig. 2, lower panel). Plasma metanephrine, normetanephrine, and chromogranin A were all normal. Urine steroid profile demonstrated increased cortisol metabolites, low androgens, and no precursor steroids. During surgery when the left adrenal was mobilized, blood pressure increased to 250/120 and i.v. alpha blockade and adenosine were administered due to suspicion of a pheochromocytoma. After surgery the patient needed norepinephrine infusion on top of the hydrocortisone infusion. Histology revealed a radically extirpated adrenal mass presenting as nodular adrenocortical hyperplasia together with adrenal medullar hyperplasia, in which 10 % of the adrenal medullary cells expressed ACTH but the Ki67-index was low (<1 %) (Fig. 3, lower panel). In contrast, there was no ACTH expression in the nodular adrenocortical hyperplasia tissue. After surgery the patient felt better and the hydrocortisone supplementation was slowly lowered. However, at the last follow-up 2.5 years later serum cortisol and plasma ACTH were still suppressed and she still needed hydrocortisone replacement, felt tired, and was working only 50 %.

## Discussion

Only 1.2 % of all patients with Cushing’s syndrome were due to ACTH secretion from a pheochromocytoma or an adrenal medullary hyperplasia, and only 1.3 % of all pheochromocytomas identified also resulted in an ectopic ACTH syndrome. This is similar to a study that identified that 1 % of all pheochromocytomas also had an ectopic hormone secretion, of which 50 % had excess cortisol secretion [6]. Of all our cases with Cushing’s syndrome, 61.6 % were due to a pituitary tumor, 32.3 % due to an adrenocortical tumor/hyperplasia (3.6 % adrenocortical cancer), and 6.1 % due to ectopic ACTH syndrome, which is in accordance with a recent review [1]. However, the frequency of Cushing’s syndrome as a result of ACTH secretion from a pheochromocytoma or adrenal medullary hyperplasia is unclear from the literature. It has been claimed that 2.5–5 % of all ectopic ACTH syndromes is caused by a pheochromocytoma [5, 10], while we found ACTH secretion in 10 % from medullary cells in a pheochromocytoma and 10 % from adrenal medullary hyperplasia. The differences may be the result of different selection biases and/or chance in these rare cases. Anyhow, an under-registration can easily occur. Most patients with ectopic ACTH syndrome are primarily seen by oncologists and as these patients are very unwell and maybe treated with glucocorticoids, makes it a challenge to suspect and detect Cushing’s syndrome. Moreover, our endocrine service may only have seen some patients as in-patient consults or been consulted on telephone. Thus, these would not been registered in our out-patient database, which may explain some of the differences. In some cases of pheochromocytoma-associated ACTH-dependent Cushing’s syndrome, the ectopically secreted hormone is CRH, with ACTH being secreted by pituitary hyperplasia [8]. Since pre-operative petrosal sinus sampling/CRH assays and post-operative CRH immunostaining are not routinely performed when pheochromocytoma-associated ACTH-dependent Cushing’s syndrome is suspected, some published cases could have been misclassified as ACTH-secreting instead of CRH-secreting.

Adrenal medullary hyperplasia can be viewed as a precursor of a pheochromocytoma and have been associated with familiar pheochromocytomas [11]. The distinction between adrenal medullary hyperplasia and pheochromocytoma is mainly based on the size of the lesion with <1 cm considered to represent hyperplasia, provided it grossly, and microscopically resembles the rest of the medulla [12]. Adrenal medullary hyperplasia is sometimes considered a benign lesion, which does not overproduce catecholamines [13]; however, others have reported mild increased catecholamine secretion and symptoms and recommend surgery with a similar preoperative preparation as with pheochromocytomas [14]. To the best of our knowledge, our case of adrenal medullary hyperplasia is the first reported with a concomitant ACTH secretion. The two cases describe the large spectrum of ectopic ACTH syndrome. The first case had in hindsight several years of symptoms from increased catecholamine production; however, severe hypercortisolism with hypokalemia developed in just over a month. Ectopic ACTH syndrome can develop very rapidly and too quickly to develop the classical signs of Cushing’s syndrome. Under such circumstances a bilateral adrenalectomy can be a life-preserving emergency therapy [15]. In contrast, the second case had a slower progression of her Cushing’s syndrome developing over several years, although she got worse the last few months before diagnosis. In this case, no biochemical evidence of a pheochromocytoma was found pre-operatively, but signs of catecholamine hyper-secretion were found during surgery. Interestingly, this case demonstrated an ACTH upregulation with paradoxical response to dexamethasone similar to what has been recently described by Sakuma et al. [16]. They presented a case where both ACTH and catecholamine secretion were upregulated by dexamethasone. With immunohistological and in vitro studies it was indicated that hypomethylation of the proopiomelanocortin promoter may be responsible for the paradoxical ACTH response to dexamethasone. Also post-operatively there were differences between the cases with the first recovering quickly, while the second had not recovered fully after almost 3 years. The second patient is still suffering from impaired quality of life and mental fatigue, a common complication in patients with Cushing’s syndrome despite remission [17].

The clinical characteristics and pathological findings of these two cases with ectopic ACTH production compared to other pheochromocytomas without ACTH production were quite evident with signs and symptoms of increased cortisol secretion, even though they did not both present with all classic signs such as hypokalemia, hyperglycemia (even though also pheochromocytomas can give hyperglycemia but normally not to the same degree), easy bruising, changes in fat distribution, and so on. Other signs such as hypertension are often found in both condition but hypertensive crisis is more associated with increased catecholamine levels similar to tachycardia and headaches. On the other hand, suspecting ectopic ACTH production from a pheochromocytoma or an adrenal medullary hyperplasia can be more challenging, since the catecholamine-related symptoms and signs may easily be obscured by the cortisol-related ones. To overcome some of these challenges and potential serious consequences, we recommend analyzing plasma metanephrines and evaluation for hypercortisolism in all adrenal tumors, irrespective if an adrenocortical or medullar tumor is suspected. However, adrenal medullary hyperplasia may still be undiagnosed illustrated by our second case.

The inherent limitations of all retrospective studies, in particularly that of ascertainment bias, are also present in this study. Moreover, not all biochemical and genetic tests together with imaging, which in hindsight would have been interesting, were done.

## Conclusion

The clinical presentation of ACTH-secreting pheochromocytomas and adrenal medullary hyperplasia can be anything from mild to dramatic. They are rare conditions but important to bear in mind in the workup of a patient with Cushing’s syndrome or with pheochromocytoma. The identification of ectopic ACTH production from adrenal medullary lesions can be life-saving.
